# Prediction of self-efficacy in recognizing deepfakes based on personality traits 

**DOI:** 10.12688/f1000research.128915.1

**Published:** 2022-12-19

**Authors:** Juneman Abraham, Heru Alamsyah Putra, Tommy Prayoga, Harco Leslie Hendric Spits Warnars, Rudi Hartono Manurung, Togiaratua Nainggolan

**Affiliations:** 1 Psychology Department, Faculty of Humanities, Bina Nusantara University, Jakarta, 11480, Indonesia; 2 Content Collision, Jakarta, 11470, Indonesia; 3 Information System Concentration, Doctor of Computer Science Department, Bina Nusantara University, Jakarta, 11530, Indonesia; 4 Japanese Department, Faculty of Humanities, Bina Nusantara University, Jakarta, 11480, Indonesia; 5 Research Center for Social Welfare, Village, and Connectivity, National Research and Innovation Agency, Jakarta, 10340, Indonesia

**Keywords:** deepfake detection, deepfake recognition, self-efficacy, personality, traits

## Abstract

**Background:** While deepfake technology is still relatively new, concerns are increasing as they are getting harder to spot. The first question we need to ask is how good humans are at recognizing deepfakes - the realistic-looking videos or images that show people doing or saying things that they never actually did or said generated by an artificial intelligence-based technology. Research has shown that an individual’s self-efficacy correlates with their ability to detect deepfakes. Previous studies suggest that one of the most fundamental predictors of self-efficacy are personality traits. In this study, we ask the question: how can people’s personality traits influence their efficacy in recognizing deepfakes? **Methods:** Predictive correlational design with a multiple linear regression data analysis technique was used in this study. The participants of this study were 200 Indonesian young adults. **Results:** The results showed that only traits of Honesty-humility and Agreeableness were able to predict the efficacy, in the negative and positive directions, respectively. Meanwhile, traits of Emotionality, Extraversion, Conscientiousness, and Openness cannot predict it. **Conclusion:** Self-efficacy in spotting deepfakes can be predicted by certain personality traits.

## Introduction

One of the biggest threats and disruptions to privacy and democracy in this digital age is deepfake technology. A ‘deepfake’ or synthetic media, is a video editing technology that manipulates and mimic a person’s facial expressions, mannerisms, voice, and inflections based on a large amount of data of other people to create a hyper-realistic video depicting them doing or saying things that never happened ( Westerlund, 2019).

The current consensus is that the average human’s ability in recognizing deepfakes is similar to the machines ( Vitak, 2022). However, the result seems to vary depending on their own confidence and belief in their cognitive abilities. Some studies suggest that some individual differences determine if a person is good at recognizing deepfakes or not ( Shahid *et al.*, 2022). In this study, we will look at the relationship between personality traits and people’s efficacy in recognizing deepfakes.

The HEXACO personality model describes six facets of personality structures: Honesty-humility, Emotionality, Extraversion, Agreeableness, Conscientiousness, and Openness to experience ( Lee & Ashton, 2009; unpublished report). Multiple studies in various contexts have shown that personality traits influence an individual’s self-efficacy ( Lodewyk, 2018).

The Honesty-humility dimension reflects an individual’s fair-mindedness, modesty, and cooperation. A person with high Honesty-humility might not think they are good at recognizing deepfakes, regardless of their true ability while an individual with low Honesty-humility might be biased in their ability in recognizing a deepfake.

Emotionality reflects an individual’s degree of anxiousness, fearfulness, and sentimentality - the experience of anxiety in response to life’s stressors. To overcome this anxiety, the sense of being able to recognize deepfakes is important to reduce that anxiety. One way to become less anxious is to appreciate deepfakes as a “cultural technology” ( Cover, 2022) that contains artistic and creative values. People with high Emotionality may be more motivated to use deepfakes as an "antidote" from the pressures of everyday life, so they have higher efficacy to detect them, not to be avoided but as potential things to be used according to their interests (technology appropriation; see Prayoga & Abraham, 2017)

Extraversion reflects an individual’s degree of sociability. Individuals high in Extraversion might have higher self-efficacy due to their higher social esteem, boldness, and familiarity. Van der Zee *et al.* (2002) found that extroverts are friendly and less formal in their interactions with others. This is closely connected with emotion recognition (part of emotional intelligence) which affects the success of negotiations. By using the paradigm of the social construction of technology ( Kwok & Koh, 2021), humans are parties who "negotiate" with technology to better recognize the technology, including deepfakes, and can adapt it to not become victims of technology—or misappropriate technology for evil interests—but rather agents who utilize technology to improve humanity and prevent harm posed by technology (such as deepfakes).

An Individual’s degree of cooperation, tolerance, flexibility, and patience is reflected in the Agreeableness dimension. More agreeable people are at a larger risk for security, and social engineers (like deepfake designers) specifically target Agreeableness attributes like benevolence and compliance.

Conscientiousness reflects precisions, cautiousness, and a degree of self-control. Individuals with higher Conscientiousness thread might have higher self-efficacy in recognizing deepfakes. This is in line with the hypothesis of Köbis *et al.* (2021) that increasing Conscientiousness will make people motivated to invest cognitive resources to detect deepfakes, thereby enhancing their capacity to recognize truth and decreasing their desire to spread false information.

Openness reflects the willingness to experience new things and is associated with lower risk aversion. Research by Uebelacker and Quiel (2014) shows that open people don’t create suitable coping mechanisms because they misjudge their vulnerability to being a target of social engineering (like deepfake designers).

## Methods

There is only one data collection stage and there is no exposure in this study.

### Ethical considerations

This present study was initially approved by the Bina Nusantara University Research Committee, vide Letter of Approval No. 042/VR.RTT/VI/2021, strengthened with Letter No. 127/VR.RTT/VI/2022. The ethical decree is stated in Article 1 Paragraph 2 of the Letter.

Written informed consent was obtained from all participants of this study, which included consent for the research procedure to be carried out and for the publication of this article containing anonymized, analyzed, and interpreted data.

The eligibility criteria of the samples were young adults aged 18–25 years (Generation Z), which, according to a YouGov survey, is an age group who are concerned about a deepfake video of themselves going viral online ( Help Net Security, 2022; unpublished report). The participants of this study were 200 young adults (139 women, 61 men; *M* = 22.06 years old; *SD* = 1.98 year) who came from a non-Western country, Indonesia, and were recruited using a convenience sampling technique. The number of sample came from a calculation using the Sample Size Calculator ( Calculator.net, 2022), with the following parameters: Confidence level of 95%, population size of 68,662,815 and population proportion of 27.94% - which was the total population of generation Z in Indonesia ( Widi, 2022; unpublished report), as well as a margin of error of 6.3% - which is still in the range of 4–8%, the acceptable one ( Cork Institute of Technology, 2020; unpublished report).

The research was conducted for 6 months from planning, participant recruitment, to data analysis. The research location is in Indonesia in an online setting for 3 months, namely 1 May to 31 July 2022. There is only one data collection stage. There was no exposure or treatment because the research was not an experimental study. The research was a cross-sectional study, so no follow-up procedure was applied.

Participants filled out an electronic questionnaire consisting of demographic data and two scales, namely HEXACO Personality Traits (as the predictors) and Self-efficacy in recognizing deepfake (as the criterion variable). The design of this study was predictive correlation.

To measure self-efficacy in recognizing deepfakes, the authors constructed a self-efficacy measuring tool based on Bandura’s theory (1977) which is combined with ways to detect deepfakes taken from an unpublished report (Johansen, 2020). The introductory question was: "How sure are you that you can recognize or detect the presence of non-original or unnatural or unnatural elements ( *e.g.* because it has been EDITED/MANIPULATED) from every image, photo, sound, and video you encounter?" Examples of items were: (1) I feel able to see abnormal eye movements; (2) I feel that I recognize awkward faces, *e.g.* if someone’s face is pointing in one direction and the nose is pointing the other way; (3) I feel able to see any inappropriate skin tone in a video; (4) I am confident of being able to recognize when a person’s face does not seem to convey the emotion that should be in line with what the person is supposed to say. There were six answer choices, ranging from "Feeling Very Incompetent" (scored 1) to "Feeling Very Capable" (scored 6).

To measure personality traits, this study used the short version of HEXACO-PI-R (60 items) (Lee & Ashton, 2009) with a scoring key. The response option ranged from “Strongly Disagree” (scored 1) to “Strongly Agree” (scored 6). The author translated the measuring tool into Indonesian.

The underlying data ( Abraham & Alamsyah, 2022a) and complete questionnaire ( Abraham & Alamsyah, 2022b) are openly available.

## Results

Demographically, some participants were residents of DKI Jakarta province ( *N*=90) which is the capital of Indonesia. In addition, other participants were residents of the Java Island (non-DKI Jakarta; *N*=86); Sumatera Island ( *N*=21); and the rest ( *N*=3) came from East Kalimantan, North Maluku, and West Nusa Tenggara provinces.

**Figure 1.**
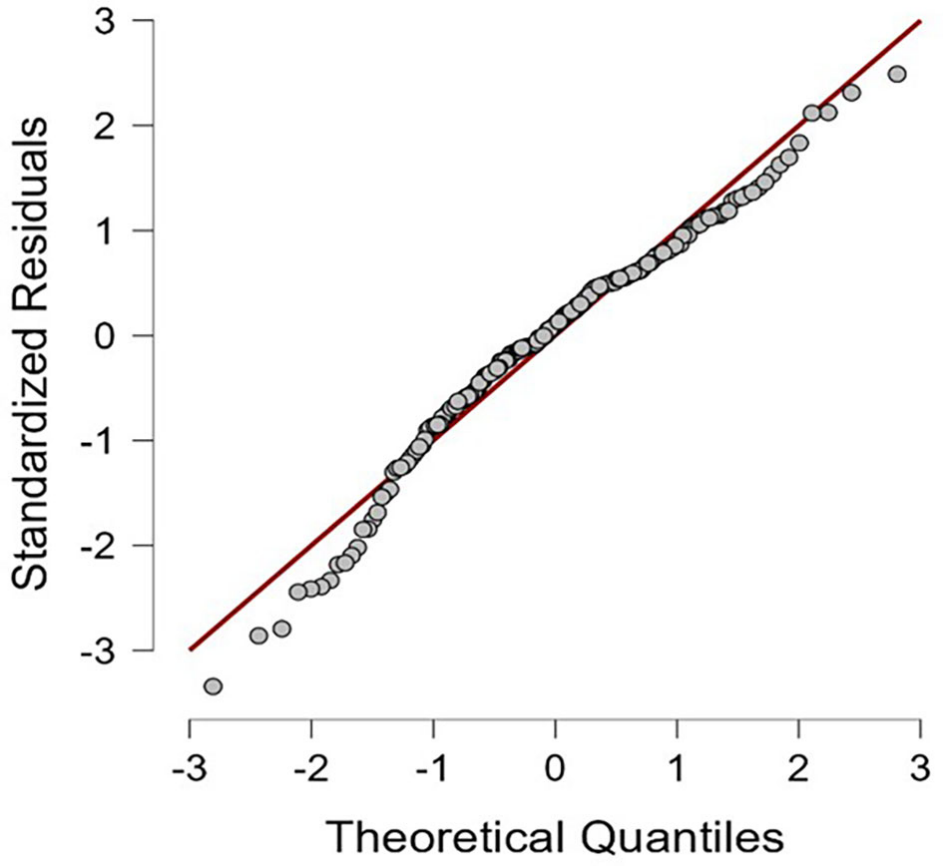
Q-Q plot standardized residuals.

The psychometric properties and descriptive statistics of the variables are shown in Table 1. The results of this study indicate that the residuals are normally distributed ( Figure 1) and all HEXACO personality dimensions are negatively correlated with self-efficacy in recognizing deepfakes; except for Agreeableness, which positively correlated (see Table 2). However, the results of the regression analysis with *F*(6,199)=13,295, *p*=0.000, *R* ^2^=0.292, showed that only Honesty-humility and Agreeableness were able to predict the efficacy (see Table 3). No difference was found between women and men, *t*(198)=−0.120, *p*=0.904, in terms of self-efficacy.

**Table 1.** Descriptives ( *N*=200).

Variable	Cronbach’s *α*	Corrected Item-Total Correlations	*n* of items [before; after validation]	*M*	*SD*	*SE*
Honesty-humility	0.851	0.534-0.723	10; 6	2.910	1.015	0.072
Emotionality	0.671	0.433-0.528	10; 3	2.680	0.952	0.067
Extraversion	0.760	0.363-0.811	10; 5	2.325	0.766	0.054
Agreeableness	0.698	0.305-0.533	10; 6	3.782	0.653	0.046
Conscientiousness	0.817	0.478-0.625	10; 6	2.664	0.894	0.063
Openness	0.729	0.472-0.619	10; 5	2.702	0.819	0.058
Self-efficacy in recognizing deepfake	0.935	0.483-0.696	23; 23	4.360	0.762	0.054

*Note.* *M* = mean, *SD* = standard deviation, *SE* = standard error

**Table 2.** Pearson’s Correlations ( *N*=200).

Variable		1	2	3	4	5	6	7
1. H	Pearson’s *r*	—						
	*p*	—						
2. E	Pearson’s *r*	0.641 ^***^	—					
	*p*	1.524e-24	—					
3. X	Pearson’s *r*	0.510 ^***^	0.378 ^***^	—				
	*p*	1.178e-14	3.511e-8	—				
4. A	Pearson’s *r*	-0.487 ^***^	-0.548 ^***^	-0.364 ^***^	—			
	*p*	2.469e-13	4.219e-17	1.132e-7	—			
5. C	Pearson’s *r*	0.740 ^***^	0.606 ^***^	0.554 ^***^	-0.443 ^***^	—		
	*p*	6.084e-36	1.965e-21	1.668e-17	5.348e-11	—		
6. O	Pearson’s *r*	0.674 ^***^	0.591 ^***^	0.460 ^***^	-0.483 ^***^	0.641 ^***^	—	
	*p*	7.910e-28	3.221e-20	7.048e-12	4.106e-13	1.713e-24	—	
7. SE	Pearson’s *r*	-0.463 ^***^	-0.367 ^***^	-0.285 ^***^	0.465 ^***^	-0.403 ^***^	-0.381 ^***^	—
	*p*	5.244e-12	9.018e-8	4.268e-5	4.229e-12	3.278e-9	2.591e-8	—

*Note.* H=Honesty-humility, E=Emotionality, X=Extraversion, A=Agreeableness, C=Conscientiousness, O=Openness, SE=Self-efficacy in recognizing deepfake, *r* = Pearson’s correlation coefficient, *p* = statistical significance of observed results

* *p* <0.05,

** *p* <0.01,

^***^ *p* <0.001

**Table 3.** Multiple linear regression predicting self-efficacy in recognizing deepfake ( *N*=200).

							Collinearity Statistics	
Model		*B*	*SE*	*β*	*t*	*p*	Tolerance	*VIF*
H _0_	(Intercept)	4.360	0.054		80.871	3.532e-154		
H _1_	(Intercept)	3.733	0.491		7.603	1.234e-12		
	Honesty-humility	-0.192	0.077	-0.255	-2.491	0.014	0.349	2.863
	Emotionality	0.023	0.070	0.029	0.332	0.740	0.475	2.104
	Extraversion	0.003	0.075	0.003	0.044	0.965	0.655	1.528
	Agreeableness	0.361	0.088	0.309	4.090	6.326e-5	0.643	1.555
	Conscientiousness	-0.068	0.085	-0.079	-0.798	0.426	0.372	2.691
	Openness	-0.026	0.083	-0.028	-0.312	0.755	0.462	2.166

*Note.* *B* = unstandardized beta, *SE *= standard error for the unstandardized beta, *β* = the standardized beta, *p* = statistical significance of observed results, *VIF* = variance inflation factor ( *VIF* < 4 reflects no multicollinearity)

Table 3 shows the unadjusted ( *B*) and adjusted ( *β*) estimates for each predictor of which the potential confounders are the personality traits dimensions other than the focused predictor.

## Discussion

This study found that the personality trait of Honesty-humility had negative predictive correlation with self-efficacy in recognizing deepfakes, *β*=-0.255, *t*(193)=-2.491, *p*<0.05 ( Table 3). “Persons with very high scores on the Honesty-Humility scale avoid manipulating others for personal gain, feel little temptation to break rules, are uninterested in lavish wealth and luxuries, and feel no special entitlement to elevated social status” (Lee & Ashton, 2009, para 1). A person’s Honesty-humility trait do not want to engineer others but, ironically, this trait makes them vulnerable to being manipulated by others ( Ternovski *et al.*, 2021), including deepfakes, especially in the context of political greediness. It can drive higher errors for the trait in recognizing deepfakes, opening vulnerabilities in security and political integrity.

This study found that Emotionality cannot predict self-efficacy in recognizing deepfakes, *β*=-0.029, *t*(193)=0.332, *p*>0.05 ( Table 3). Austin and Vahle (2016) found that Emotionality—a trait that is positively correlated with empathy and social engagement—can predict the dimensions of Enhance (providing support and reassurance as interpersonal emotion management strategies) and Divert (the practice of using humor and pleasure pursuits to lift the spirits of others) of the Managing the Emotion of Others Scale (MEOS). This means that the Emotionality dimension is also positively correlated with the emotional intelligence needed to recognize deepfakes. Yang *et al.* (2022) emphasized the pivotal role of emotional intelligence in improving artificial intelligence technology so that it becomes a useful deepfake in the context of clinical encounters. By knowing that deepfakes themselves are increasingly being prepared with elements of emotional intelligence, then recognizing deepfakes also requires a better one; and this intelligence can actually be found in people with higher Emotionality. However, individuals high in Emotionality might be less confident in their own ability to accurately recognize deepfakes, as they might consider more factors and doubt themselves more ( Thompson, 1998). With this uncertain direction, it is not surprising that no predictive power of Emotionality was found on self-efficacy.

This study found that Extraversion is a personality trait that cannot predict self-efficacy in recognizing deepfakes, *β*=0.003, *t*(193)=0.044, *p*>0.05 ( Table 3). Hosler *et al.* (2021) put forward that detecting deepfakes is actually recognizing unnatural displays of emotion in voices and faces. Emotion apparently plays a central role in recognizing deepfakes because emotion is a higher-level semantic construct—which is difficult to counterfeit up to now—that could offer hints for detection. In an unpublished report, Kill states that emotion recognition is an ability that is honed in someone with a high extraversion trait ( 2021). However, Extraversion is also found to be positively correlated with excitement-seeking and a lower preference for consistency ( Uebelacker & Quiel, 2014) - whereas “pairwise self-consistency learning” ( Zhao *et al.*, 2021, p. 15023) is needed to recognize deepfakes. Therefore, the effects of Extraversion traits appear to cancel out of each other resulting in no predictive correlation with the self-efficacy.

This study found that the Agreeableness trait can predict self-efficacy in recognizing deepfakes; however, not as hypothesized, the direction was found positive – not negative, *β*=0.309, *t*(193)=4.090, *p*<0.05 ( Table 3). People with high Agreeableness are eager to cooperate and reach a compromise with others (Lee & Ashton, 2009). One of the good “others” in the context of deepfake recognition or detection is the “wisdom of the crowds” ( Groh *et al.*, 2022), which Surowiecki (2004) defines as “the collective intelligence that arises when our imperfect judgments are aggregated”. Agreeing with (or high Agreeableness to) the collective intelligence should reduce the chance of falsely recognizing deepfakes, including its algorithm attempts that present visual obstructions such as misalignment, partial occlusion, and inversion.

This study found that Conscientiousness was not able to predict self-efficacy in recognizing deepfakes, *β*=-0.079, *t*(193)=-0.798, *p*>0.05 ( Table 3). Although deepfake recognition requires conscientious characteristics such as prudence and a sense of responsibility, Lawson and Kakkar’s (as cited in Sütterlin *et al.*, 2022) research recently found that Conscientiousness is partially correlated with belief in conspiracy and conservatism - making it less efficacious in recognizing deepfakes.

This study found that Openness was not able to predict self-efficacy in recognizing deepfakes *β*=-0.028, *t*(193)=-0.312, *p*>0.05 ( Table 3). In an unpublished report, Jin (2020) found that values of Openness to change do not correlate with the perceived ethical implications of deepfakes ( *e.g.*, "These videos can uncontrollably deceive and influence many people", p. 24). In addition, contrast with the certain direction of the influence of Agreeableness and Honesty-humility on the self-efficacy; the direction of the Openness prediction is ambiguous. On the one hand, Openness is related to the low ability to recognize deepfakes. It is because Openness was found to be positively correlated with cognitive ability ( Curtis *et al.*, 2015; Rammstedt *et al.*, 2016), but cognitive abilities encourage more protective online behavior, indicated by more interest in discussing how people who use deepfakes manipulate their audiences - rather than developing ability to apply scepticism on the authenticity of videos ( Ahmed, 2021). On the other hand, there is a logic in favor of Openness as a buffer to prevent vulnerabilities from being manipulated by social engineering. For example, Eftimie *et al.* (2022) associated Openness with cognitive exploration tendencies which, based on their study, will stimulate responsible behavior including security best practices - which in the context of this study is deepfake recognition.

The limitation of this research is the use of non-probability sampling with limited generalizability. Nevertheless, this study has implication for the development of psychoinformatics - a branch of psychology that explains attitudes, competencies, and behavior in using information technology. Further research is suggested to implement random sampling and experimental methods to ensure a causal–not only predictive–relationship between personality traits and deepfakes detection self-efficacy.

## Data Availability

Zenodo: Dataset of Prediction of Self-efficacy in Recognizing Deepfake based on Personality Traits. https://doi.org/10.5281/zenodo.7357400 ( Abraham & Alamsyah, 2022a). The project contains the following underlying data:
- Dataset of Prediction of Self-efficacy in Recognizing Deepfake based on Personality Traits.xlsx (Raw data) Data are available under the terms of the Creative Commons Attribution 4.0 International license (CC-BY 4.0). Zenodo: Questionnaire of Prediction of Self-efficacy in Recognizing Deepfake based on Personality Traits. https://doi.org/10.5281/zenodo.7413517 ( Abraham & Alamsyah, 2022b). The project contains the following extended data:
- Questionnare-HEXACO and Self-efficacy.docx Data are available under the terms of the Creative Commons Attribution 4.0 International license (CC-BY 4.0).
