# Supplementary material for: Prediction of self-efficacy in recognizing deepfakes based on personality traits 
Source: F1000Res. 2023 Oct 12;11:1529. Originally published 2022 Dec 19. [Version 3] doi: 10.12688/f1000research.128915.3 (PMC10719557; doi:10.12688/f1000research.128915.3)
Supplement: Peer review history

# Reviewer report: Reviewer response for version 3

**DOI:** 10.5256/f1000research.157245.r214616

**Published:** 2023-12-13

**Authors:** Sandra Grinschgl

**Affiliations:** 1 Institute of Psychology, University of Graz, Graz, Austria

The authors revised their manuscript based on my suggestions. I still think, however, that some of the interpretations and conclusions could be softened and put in less causal way.

Is the work clearly and accurately presented and does it cite the current literature?

Partly

If applicable, is the statistical analysis and its interpretation appropriate?

Yes

Are all the source data underlying the results available to ensure full reproducibility?

Partly

Is the study design appropriate and is the work technically sound?

Yes

Are the conclusions drawn adequately supported by the results?

Yes

Are sufficient details of methods and analysis provided to allow replication by others?

Partly

Reviewer Expertise:

Human-technology interaction, personality psychology, cognitive psychology

I confirm that I have read this submission and believe that I have an appropriate level of expertise to confirm that it is of an acceptable scientific standard.

# Reviewer report: Reviewer response for version 2

**DOI:** 10.5256/f1000research.152590.r185694

**Published:** 2023-09-14

**Authors:** Dilrukshi Gamage

**Affiliations:** 1 Department of Innovation Science, Tokyo Institute of Technology, Tokyo, Japan

In summary, authors have used HEXACO Personality Traits as the independent variable and  the criteria used in Self-efficacy in recognizing deepfake. The 6 factors of the HEXACO were used to understand the self efficacy of recognizing deepfakes in a 6 point likert scale. I believe the dependent was the self-efficacy in recognizing deepfakes, where the authors constructed a self-efficacy measuring tool based on Bandura’s theory. And this was more adopted using the Notion company criteria. 

 Overall, authors have significantly improved the article, in terms of the clarity in explaining the methods and evaluations. 

 I would recommend not to explain the methods first with “There is only one data collection stage. There is no exposure in this study because the research was not an experimental study.” Although as a reviewer I know you may be addressing a comment, but if this is published, the readers have no clue on it. Explain the method first and then to be clear its a survey questionnaire . 

 I would also recommend to add a conclusion part to the manuscript summarizing the key takes aways - why some traits were removed and what implications does it reflect to the predicted efficacy. Also add the overall implication of such predictors to the efficacy items.

Is the work clearly and accurately presented and does it cite the current literature?

No

If applicable, is the statistical analysis and its interpretation appropriate?

Partly

Are all the source data underlying the results available to ensure full reproducibility?

No

Is the study design appropriate and is the work technically sound?

Partly

Are the conclusions drawn adequately supported by the results?

No

Are sufficient details of methods and analysis provided to allow replication by others?

No

Reviewer Expertise:

Computational social science, deepfakes social implications

I confirm that I have read this submission and believe that I have an appropriate level of expertise to confirm that it is of an acceptable scientific standard.

# Reviewer report: Reviewer response for version 2

**DOI:** 10.5256/f1000research.152590.r185695

**Published:** 2023-07-17

**Authors:** Sandra Grinschgl

**Affiliations:** 1 Institute of Psychology, University of Graz, Graz, Austria

This is a revised version of a manuscript that I previously reviewed. The authors addressed my previously raised comments and improved their manuscript.

 Here are a few comments that the authors might still want to consider:

1. The authors now included a brief paragraph on their hypotheses, however, to me those seem rather unspecific. In what direction would each personality trait predict self-reported efficacy in identifying deep fakes? How could the traits together predict variance in self-reported efficacy in identifying deep fakes? A related question: Why did the authors not have hypotheses for the correlational analyses?
2. Some of the interpretations in the discussion seem a bit too extreme and could be downscaled. For instance: “Generation Z with trait Honesty-Humility feels helpless, so it is less functional in detecting deepfakes.” I don’t think the authors can draw this conclusion as they did not measure the actual ability to detect deepfakes.
  “Therefore, the effects of Extraversion traits appear to cancel out of each other resulting in no predictive correlation with the self-efficacy” -> this should be formulated softer as it’s only a suggestion.

Is the work clearly and accurately presented and does it cite the current literature?

Partly

If applicable, is the statistical analysis and its interpretation appropriate?

Yes

Are all the source data underlying the results available to ensure full reproducibility?

Partly

Is the study design appropriate and is the work technically sound?

Yes

Are the conclusions drawn adequately supported by the results?

Yes

Are sufficient details of methods and analysis provided to allow replication by others?

Partly

Reviewer Expertise:

Human-technology interaction, personality psychology, cognitive psychology

I confirm that I have read this submission and believe that I have an appropriate level of expertise to confirm that it is of an acceptable scientific standard, however I have significant reservations, as outlined above.

# Reviewer report: Reviewer response for version 1

**DOI:** 10.5256/f1000research.141554.r162152

**Published:** 2023-02-20

**Authors:** Dilrukshi Gamage

**Affiliations:** 1 Department of Innovation Science, Tokyo Institute of Technology, Tokyo, Japan

**Overall, the objective of this brief report is to find out the personality traits that affect the efficacy of spotting deepfakes. **

- **Is the work clearly and accurately presented and does it cite the current literature?**

Since some of the literature cited are unpublished reports, I am not sure of the credibility. Since those were absorbed to the main study, this is pretty questionable.

- **Is the study design appropriate and does the work have academic merit?**

As mentioned in the introduction, the authors have taken the personality traits HEXACO from an  unpublished report in 2009. I am curious what the authors consider other personality traits and also why did not cite any peer reviewed article with verified and validated factors. On the other hand, introduction does not provide a smooth understanding to why such model was selected and how others have conducted such explanations. 

- **Are sufficient details of methods and analysis provided to allow replication by others?**

Methods were illustrated in a very awkward pattern, for example at once I was not sure doing such a sample size for what- the method of data collection, it took a while to understand that this is a questionnaire, and authors' explanation of other statements made this bit complicated than a straight forward mention. 

 The survey questionnaire was unclear - as  I understand it's a 6 point Likert scale, but I am not sure if the authors show any deepfake video before the question asked, and if so what are those. 

- **If applicable, is the statistical analysis and its interpretation appropriate?**

The authors were not clear upfront about their analysis structure or procedure. I believe this was not pre-registered as well. In the results Authors explain the regression analysis and also correlation coefficient of the variables (personality traits), but due to the fact that this was not explain or mentioned as hypothesis, it is not clear the objective of the results and the strategy it provide to show the evidence.

- **Are all the source data underlying the results available to ensure full reproducibility?**

Data seems to be available.

- **Are the conclusions drawn adequately supported by the results?**

The authors do not provide any concrete conclusions as per se but, the discussion is somewhat leaning toward their conclusions. 

 But I have many issues with the way the study is conducted and how authors could claim statement as follow - “Honesty-humility trait do not want to engineer others but, ironically, this trait makes them vulnerable to being manipulated by others (Ternovski et al., 2021), including deepfakes, especially in the context of political greediness.” ---> how did you think of political context, because the study did not support any assumption? Did you ask this question?

Is the work clearly and accurately presented and does it cite the current literature?

No

If applicable, is the statistical analysis and its interpretation appropriate?

Partly

Are all the source data underlying the results available to ensure full reproducibility?

No

Is the study design appropriate and is the work technically sound?

Partly

Are the conclusions drawn adequately supported by the results?

No

Are sufficient details of methods and analysis provided to allow replication by others?

No

Reviewer Expertise:

Computational social science, deepfakes social implications

I confirm that I have read this submission and believe that I have an appropriate level of expertise to state that I do not consider it to be of an acceptable scientific standard, for reasons outlined above.

# Reviewer report: Reviewer response for version 1

**DOI:** 10.5256/f1000research.141554.r160499

**Published:** 2023-02-10

**Authors:** Sandra Grinschgl

**Affiliations:** 1 Institute of Psychology, University of Graz, Graz, Austria

Summary: This article deals with a highly relevant topic - the identification of deepfakes. The authors investigated potential predictors of the self-reported ability to detect deepfakes, namely individuals’ personality traits based on the HEXACO model. While the traits honesty-humility and agreeableness were indeed shown to be predictors for self-reported ability to identify deepfakes, emotionality, extraversion, conscientiousness and openness were not. A higher honesty-humility was related to a lower self-reported ability to detect deepfakes whereas a higher agreeableness was related to a higher self-reported ability to detect deepfakes.

 I think this is an interesting article on a very acute topic, however, I think it would benefit from some revisions. I outline my concerns below.

 Major comments:

- Regarding the clear and accurate presentation of the authors’ work: In my opinion, the introduction is lacking an elaborate justification for testing the HEXACO traits as potential predictors of the (self-reported) ability to detect deepfakes. Why might especially those traits act as predictors? The authors could, for instance, argue that typical personality traits might also be relevant when it comes to the application of other artificial intelligence technologies (e.g., robots). For a reference on this behalf see Matthews et al. (2021) ^1^.

- The introduction does not end with clear hypotheses, thus, to me it is unclear whether this research was exploratory or confirmatory.

- Table 1: Why was the number of items reduced for the HEXACO traits? What does “after validation” refer do? This choice of method might potentially influence the interpretation of results.

- I think the discussion would benefit from a paragraph that focuses on the bigger picture in which the results can be placed. For instance, it could be discussed what conclusions we can draw from the study findings when it comes to the increasing distribution of deepfakes. What might be the “optimal” personality to not fall for deepfakes? Also, I think the authors should discuss how self-reported abilities in detecting deepfakes might be related to actual abilities in detecting deepfakes. This seems especially relevant as individuals’ self-estimation is not always accurate (e.g., above-average effect, Dunning-Kruger effect).

Minor comments:

  

- Page 3: “The current consensus is that the average human’s ability in recognizing deepfakes is similar to the machines (Vitak, 2022).” I’m not sure what this exactly means. Does it mean that artificial intelligence technologies analyzing videos/pictures are as good as humans in identifying deepfakes from real videos?

- Only within the methods section it became fully clear to me that the authors did not actually measure participants’ ability to detect deepfakes but rather participants’ self-reported ability to do so. I would recommend the authors to refer to “self-reported efficacy in recognizing deepfakes” already from the beginning of their article (and in the abstract) to avoid any confusion.

- Figure 1: I guess this graph shows the residuals for the multiple regression model. Please clearly state this in the figure description and corresponding manuscript text.

- Please report an effect size with the t-Test statistics on gender differences.

- I think especially the discussion could need some language-editing. Every paragraph starts with “This study found” which does not induce an optimal reading flow.

- Regarding reproducibility: Data of this study are already openly available (great!). I would suggest that the authors also make their analyses scripts available.

Is the work clearly and accurately presented and does it cite the current literature?

Partly

If applicable, is the statistical analysis and its interpretation appropriate?

Yes

Are all the source data underlying the results available to ensure full reproducibility?

Partly

Is the study design appropriate and is the work technically sound?

Yes

Are the conclusions drawn adequately supported by the results?

Yes

Are sufficient details of methods and analysis provided to allow replication by others?

Partly

Reviewer Expertise:

Human-technology interaction, personality psychology, cognitive psychology

I confirm that I have read this submission and believe that I have an appropriate level of expertise to confirm that it is of an acceptable scientific standard, however I have significant reservations, as outlined above.

## References

[1] : Evolution and revolution: Personality research for the coming world of robots, artificial intelligence, and autonomous systems. *Personality and Individual Differences* .2021;169: 10.1016/j.paid.2020.109969 10.1016/j.paid.2020.109969
